# The effect of yogurt co‐fortified with probiotic and vitamin D on lipid profile, anthropometric indices and serum 25‐hydroxi vitamin D in obese adult: A Double‐Blind Randomized‐ Controlled Trial

**DOI:** 10.1002/fsn3.1996

**Published:** 2020-11-10

**Authors:** Shima Hajipoor, Azita Hekmatdoost, Mansour Rezaei, Seyed Mostafa Nachvak, Meysam Alipour, Sodabeh Eskandari, Roghayeh Mostafai, Mohammad Reza Sobhiyeh, Reza Mohammadi, Yahya Pasdar

**Affiliations:** ^1^ Nutritional Sciences Department School of Nutritional Sciences and Food Technology Kermanshah University of Medical Sciences Kermanshah Iran; ^2^ Department of Clinical Nutrition School of Nutrition Sciences & Food Technology Shahid Beheshti University of Medical Sciences Tehran Iran; ^3^ Department of Biostatistics School of Health, Social Development and Health Promotion Research Center Research Institute for Health, Kermanshah University of Medical Science Kermanshah Iran; ^4^ Alimentary Tract Research Center, Ahvaz Jundishapur University of Medical Sciences Ahvaz Iran; ^5^ Vascular and Endovascular Surgeon, Department of Surgery, Imam Reza Hospital Kermanshah University of Medical Sciences Kermanshah Iran; ^6^ Department of Food Science and Technology School of Nutrition Sciences and Food Technology Kermanshah University of Medical Sciences Kermanshah Iran

**Keywords:** lipid profile, obesity, probiotic‐fortified yogurt, vitamin D deficiency, vitamin D‐fortified yogurt

## Abstract

Vitamin D deficiency can be regarded as one of the overgrowing health problem in all of the world. Evidence from a clinical trial suggested a role for probiotic bacteria in increasing vitamin D. However, probiotic's effect is strain specific and this effect should be confirmed about different strains. The objective was to determine if yogurt fortification with probiotic bacteria, *Lactobillus acidophilus* La‐B5, *Bifidobacterium lactis* Bb‐12 either alone or in combination with vitamin D can be a complementary treatment for vitamin D deficiency_._ The end‐points were vitamin D, cardio metabolic lipid profile, anthropometric indices (weight, height, waist, hip, fat mass, lean body mass) and dietary intake. A 10‐week parallel‐group, double‐blind, randomized and controlled trial was conducted on 140 obese men and women. The participants were randomly allocated to receive 100 grams either 1) plain low‐fat yogurt or 2) probiotic yogurt or 3) vitamin D‐fortified yogurt or 4) probiotic and vitamin D cofortified yogurt. All groups received low‐calorie diet. Vitamin D increased significantly in group 4 (*p* = .008), group 3 (*p* = .001) and group 1 (*p* = .012 with no difference between groups. Vitamin D‐fortified yogurt had the most effect size and showed a significant difference versus plain (*p* = .018) and probiotic yogurt (*p* = .002). Regarding lipid profile, there were no significant differences between groups. Data from this study does not support the hypothesis that yogurt fortified with probiotic bacteria, *Lactobillus acidophilus* La‐5 and *Bifidobacterium lactis* Bb‐12 either alone or in combination with vitamin D might impose any increasing effect on serum level of vitamin D in comparison with vitamin D‐fortified yogurt.

## Graphical abstract

A 10‐week parallel‐group, double‐blind, randomized and controlled trial was conducted on 140 obese men and women. The participants were randomly allocated to receive either 1) plain low‐fat yogurt or 2) probiotic yogurt or 3) vitamin D‐fortified yogurt or 4) probiotic and vitamin D co‐fortified yogurt. All groups received low‐calorie diet. vitamin D increased significantly in group 4 (*p* = 0.008), group 3 (*p *= 0.001) and group 1 (*p *= .012 with no difference between groups. Vitamin D‐fortified yogurt had the most effect size and showed a significant difference vs. plain (*p* = 0.018) and probiotic yogurt (*p* = 0.002).

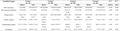

## 1 INTRODUCTION

Poor vitamin D status has become one of the most prevalent health problem in all phases of life in almost every region of the world (Li & Zhou, 2015; Ly et al., 2012). There is an ever‐increasing body of evidence that vitamin D deficiency is not only pertinent to decrease intestinal calcium resorption, but also it underlines the etiology of several metabolic disorders (Avastano, Barrea, Savanelli, 2017).

Currently, obesity is the fifth greatest risk factor for mortality (Pereira‐Santos et al., 2015). Apart from psychological stresses including body image, disparagement, impairment of quality of life and depression, which as a result impose a huge financial burden on health care system; it is well known that individuals with obesity are at greater risk for numerous medical conditions such as chronic and life‐threatening disorders including type 2 diabetes, hypertension, cardiovascular disease, hyperlipidemia, sleep apnea (Kadooka et al., 2010).

There is an ongoing debate that vitamin D deficiency is a consequence of obesity or one of its risk factors. It is generally accepted that, obese people have lower 1,25 (OH)_2_ D_3_ in comparison to normal weight people. Moreover, it has been indicated that serum 1,25(OH)_2_D_3_ is inversely correlated with body weight, BMI, and fat mass (Walsh, Bowles, Evans, 2017). Several mechanisms have been proposed for the lower 1,25(OH)_2_ D_3_ in obese people (Pourshahidi, 2015; Wamberg et al., 2015). The most discussed mechanism is that in obese people, vitamin D is distributed into greater fat volume rather than normal weight people (Pourshahidi, 2015). On the other hand, it has been proposed that differentiation of adipocytes is halted by both 1,25 (OH)_2_ D and VDR (Li & Zhou, 2015; Marcotorchino et al., 2014). Moreover, an association between adiposity phenotypes and vitamin D receptor (VDR) gene polymorphisms has been implied in numerous studies (Sollid et al., 2016).Therefore, vitamin D deficiency could result in fat augmentation which deteriorated clinical status of obesity (Walsh et al., 2017). It has recently been proposed that vitamin D deficiency may further be associated with dysbiosis of the gut microbiota which has been previously considered as a key risk factor for obesity (Ly et al., 2012; Sanz et al., 2013). Also, vitamin D receptor (VDR) is of critical importance in the regulation of intestinal homeostasis by preventing pathogenic bacterial invasion, inhibiting inflammation, and maintaining cell integrity (Kong et al., 2008; Yoon & Sun, 2011). On the other hand, manipulation of gut flora composition by delivering probiotic bacteria either as isolated bacteria or in food that has been enriched by probiotics can be regarded as an attractive treatment strategy of obesity management (Mollakhalili et al., 2017; Rouhi et al, 2015; Vallianou et al., 2020).

Growing evidence now supports the integrated relationship between obesity progress and poor vitamin D status and gut flora composition. It is noticeable that more recently, a study performed on hypercholesterolemic adults showed an increased circulating 25‐hydroxyvitamin D in response to oral probiotic supplementation (Jones et al., 2013). However, this study was the first report showing this effect and should be confirmed by a well‐designed study to distinguish the effect of probiotics on vitamin D absorption. To address this gap, our study was designed to evaluate the effect of probiotics on vitamin D status.

## 2 MATERIAL AND METHOD

### 2.1 Study design and population

This study was a parallel‐group, double‐blinded, randomized and controlled trial. One‐hundred and forty volunteers aged 20–60 years (40 men and 100 women) were selected between November and December 2018 and recruited through advertisement in public places and social media. Inclusion criteria included the following: body mass index (BMI) >30, no antibiotic therapy for the last 1 month, no metabolic disease (including: diabetes, cancer and kidney disease), no intake of probiotic product 1 month before the study, no anemia, no intake of vitamin D for 1 month before the study initiation. Furthermore, pregnant, menopause and lactating women could not participate in study. Volunteers signed written consent form to participate in the intervention.

**Figure 1:**
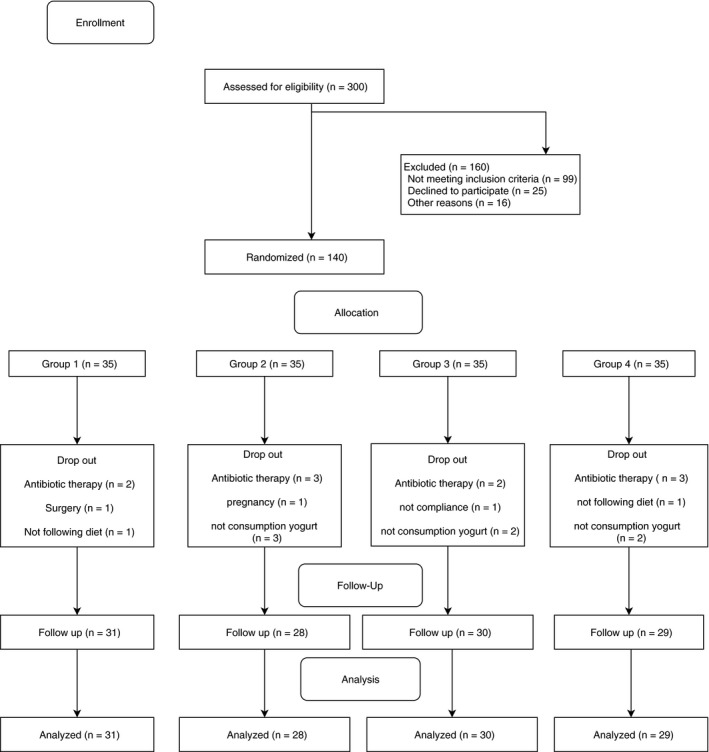
Flow diagram of study

The follow‐up diagram is shown in Figure 1. Participants were allocated by block randomization, with a block size of four to either 1) regular low‐fat yogurt plus a low‐calorie diet, 2) probiotic yogurt plus a low‐calorie diet, 3) vitamin D‐fortified yogurt plus a low‐calorie diet and 4) probiotics and vitamin D‐ fortified yogurt plus a low‐calorie diet. Random assignment was done by an independent researcher who was not involved in the data collection, analysis or reporting performed. All subjects and main investigator remained blinded until after analysis of results. There was a significant difference between groups at the baseline concerning some anthropometric factors (WC, BMI, protein and fat intake). No change was made in method, inclusion and exclusion criteria after trial commencement. Primary study outcomes included vitamin D serum concentration and lipid profile (LDL, HDL, TC and TG). Secondary study outcomes included, anthropometric factors. All of the outcomes were measured at the baseline and after 10 weeks.

### 2.2 Yogurt

Probiotic yogurt contained *Lactobacillus Acidophilus* La‐B5 and *Bifidobacterium lactis* Bb‐12 (at levels of 4 × 10^7^ colony‐forming units (CFU) of each strains). Furthermore, for preparing vitamin D‐fortified yogurt, 1,000 international unit (IU) of 1,25 (OH)_2_ D_3_ was added. Probiotic and vitamin D cofortified yogurt contained 1,000 IU vitamin D and 4 × 10^7^ CFU/ml of two aforementioned probiotic strains. All participants received the same amount of yogurts (100 grams per day) and the yogurts were the same regarding taste, texture, composition and color. Nutritional characteristics and ingredients of yogurt are showed in table 1.

**Table 1:** Nutritional characteristics and ingredients of yogurt

Energy (kcal/gram)	Carbohydrate (g)	Protein (g)	Fat (%)	Na (mg)	Probiotics (CFU/ml)	Vitamin D (IU)
53.6	6.2	4.5	1.2	108	4 × 10^7^	1,000

Abbreviation: Na, sodium.

### 2.3 Diet

All the intervention groups and plain yogurt group participated in diet therapy sessions and were received the same diet. Calorie restriction was done based on a diet program including 500–1000 kcal deficit to the baseline energy. To ensure subjects compliance all subjects were asked to record their food intake for 3 days which represents their usual food intake at baseline and at the end of intervention (one weekend day and 2 weekdays). The data were analyzed by the use of Nutrition 4 software. To reduce the all possible errors in reporting the dietary intake all participants were taught how to count their calorie intake in the same sessions. Moreover, subjects were being followed up by telephone calls.

### 2.4 Physical activity

The intervention program included recommendation to increase activity as much as 45–60 min’ moderate activity 3 times a week. All subjects were asked to not participate in any intense activity and obey the protocol. Therefore, shortened form of international physical Activity Questionnaire (IPAQ) was used to calculate Total Metabolic Equivalent (MET) of task (hours per week) was calculated to estimate daily activity before and after the intervention (Mousa et al., 2017). It includes seven questions related to physical activity associated with work, homework, and leisure time respectively during the past seven days. The questionnaire had previously been validated in Iran.

### 2.5 Sun exposure

Our study was done in winter (December‐ February) which sun light and as a result sun exposure is at the minimum of it amount, however, since it can affect amount of serum level of vitamin D even slightly, we consider it averagely by conducting a self‐report questionnaire. All subjects have been asked to estimate how many hours per day they have been under the sun exposure averagely.

### 2.6 Biochemical measurement

Serum Vitamin D was measured by using Monobind vitamin D kit (Monobind Inc). Lipid profile (TG, TC, HDL) was measured by Monobind kit (Monobind Inc). LDL was calculated by Friedewald equation = TC − HDL − 0.2 × TG (Friedewald et al., 1972).

### 2.7 Anthropometric measurement

All of the anthropometric factors including weight, height, body mass index (BMI), waist circumference (WC), hip circumference (HC), waist hip ratio (WHR) and body composition.

### 2.8 Blood sampling

All participants were asked to attend the laboratory after an overnight fast (12 hr). A 10 ml of blood was taken from each participants before and after the intervention. To obtain serum, sample was centrifuged, then serum was separated in microtube and stored at −40°C.

### 2.9 Data Analysis

At first, the normality of data was analyzed by Kolmogorov–Smirnov and chi‐square. Within group changes were assessed by Wilcoxon and independent *t*‐test. Between‐group difference was assessed by Kruskal–Wallis. This data was analyzed using SPSS ver.23 software. Moreover, to calculate the mean difference of intervention effect on main outcomes between groups, we used the effect size (EE) equation = 100* P_1_‐P_2_/P_1_ (p indicates parameter). Furthermore, the difference between groups was assessed by Kruskal–Wallis.

## 3 RESULT

### 3.1 Baseline date

From 140 individuals who were interested in participating in the study, 118 subjects completed the 10‐wk intervention (Figure 1). After starting the intervention, a total of 22 subjects dropped out, including 2 participants who could not continue the study because of pregnancy and surgery, 10 subjects due to starting antibiotic therapy, 7 subjects because they had not consumed the yogurt regularly. Furthermore, 2 subjects were excluded because they had not adhered to the diet; in addition, 1 subject did not wish to continue the study because of noncompliance. Baseline characteristics of all subjects are shown in Table 2. At baseline, there were no statistically significant differences in demographic data including age, gender, age, marital status and education and physical activity and sun exposure (Table 2). There was a significant difference in some physical measurement's mean including: waist circumference (*p* = .010) and BMI (*p* = .027) between the intervention groups. Furthermore, there were significant differences in mean of protein (*p* = .012) and fat (*p* = .001) intake between groups.

**Table 2:** Baseline characteristics of groups

	Variable/yogurt	Group 1 (*n* = 31)	Group 2 (*n* = 28)	Group 3 (*n* = 30)	Group 4 (*n* = 30)	*p*‐Value
Marital status	Single	23 (26.7)	20 (23.3)	19 (22.1)	24 (27.9)	.472*
	Married	8 (24.2)	8 (24.2)	11 (33.3)	6 (18.2)	
Education	Under diploma	23 (24)	23 (24)	25 (26)	25 (26)	.766*
	Diploma	8 (34.8)	5 (21.7)	5 (21.7)	5 (21.7)	
Vitamin D status (female)	Sever deficiency	2 (16.7)	2 (16.7)	6 (50)	2 (16.7)	.069*
	Deficiency	12 (32.4)	8 (21.6)	7 ( 18.9)	10 (27)	
	Sufficient	11 (30.6)	10 (27.8)	8 (22.2)	7 (19.4)	
Vitamin D status (male)	Sever deficiency	0 (0)	1 (33.3)	2 (66.7)	0 (0)	.172*
	Deficiency	3 (21.4)	4 (23.5)	4 (23.5)	6 (35.3)	
	Sufficient	3 (21.4)	3 (21.4)	3 (21.4)	5 (35.7)	
Age		35.37 ± 11.69	40.9 ± 6.75	48.36 ± 9.70	36.35 ± 21.10	.902**
vitamin D status < 36	Sever deficiency	2 (20)	1 (10)	6 (60)	1 (10)	.463*
	Deficiency	8 (26.8)	6 (21.4)	3 (10.7)	11 (39.3)	
	Sufficient	7 (29.2)	6 (21.4)	6 (25)	5 (20.8)	
Vitamin D status>=36	Sever deficiency	0 (0)	2 (40)	2 (40)	1 (20)	.375*
	Deficiency	7 (26.9)	6 (23.1)	8 (30.8)	5 (19.2)	
	Sufficient	7 (26.9)	7 (26.9)	5 (19.2)	7 (26.9)	

Note

Values are expressed as mean ± *SD* or number (percent).

Group 1: plain yogurt, Group 2: probiotic yogurt, Group 3: vitamin D‐fortified yogurt, Group 4: probiotic and vitamin D co‐fortified yogurt.

Abbreviation: MET, Metabolic equivalent task‐hours/day.

* Stands for significance of between‐group difference obtained from Chi‐square test.

** Stands for significance of between‐group difference obtained from ANOVA.

### 3.2 Anthropometric factors

The result of anthropometric factors before and after the intervention is shown in Table 3. All of the anthropometric factors except TBW was decreased significantly in all groups, without any significant difference between groups. It seems that these changes might be due to beneficial effect of low‐calorie diet. Moreover, comparison changes between groups did not show any significant difference. Furthermore, after controlling for the confounding effects of BMI, WC, fat and protein, the results did not change significantly.

**Table 3:** Anthropometric factors of participant before and after the intervention

Variable/Yogurt	Group 1 (*n* = 31)		Group 2 (*n* = 28)		Group 3 (*n* = 30)		Group 4 (*n* = 30)		P‐V*	P‐V**
	Before	After	Before	After	Before	After	Before	After	Before	After
BF	36.57 ± 6.04	35.19 ± 7.73^a^	38.52 ± 6.04	37.23 ± 6.91^a^	35.05 ± 6.09	34.39 ± 6.90^a^	34.08 ± 5.53	32.85 ± 5.39^a^	0.052	0.116
PBF	41 (38.50–42.80)	40 (37.47–42.22)^b^	40.25 (36.37–42.70)	40.15 (36.07–42.42)^a^	40.60 (37.40–42.80)	39.40 (36.97–41.52)^a^	38.65 (33.70–42.72)	37.90 (32.15–41.72)^a^	0.329	0.472
TBW	39.24 ± 5.01	39.13 ± 4.93	42.03 ± 8.28	41.64 ± 8.19	38.55 ± 7.40	38.98 ± 6.87	40.12 ± 6.92	39.77 ± 7.02	0.390	0.451
WHR	0.93 (0.91–0.97)	0.92 (0.90– 0.96)	0.95 (0.90–0.98)	0.94 (0.89–0.97)	0.94 (0.90–1)	0.93 (0.89–1)	0.94 (0.90–0.98)	0.92 (0.90–0.98)	0.832	0.744
BMI	34.60 (31.90–38)	33.65 (30.72–37.37)^a^	36.30 (33.50–39.50)	35.70 (32.20–38.22)^a^	34.90(31.95–35.95)	33.80 (31.32–35.75)^a^	33.80 (31.35–35.05)	33.25 (30.80–34.50)^a^	0.027	0.059
LBM	54.10 (49.20–60)	54.25 (49.37–58.52)	54.55 (50.80–65)	55 (49.70–63.85)^a^	49.50 (47.25–60.55)	50.20 (47.15–60.40)	55 (49.50–61.75)	54.70 (48–61.62)	0.620	0.440
Weight	90.20 (83.7–97.50)	90.20 (80.90–97.50)^a^	93.60 (87.72–106.80)	91.15 (83.−107.85)^a^	87.30 (79.57–97.75)	86.10 (78.57–94.17)^a^	89.70 (84.05–95.52)	88.05 (81.15–95.15)^a^	0.123	0.377
WC	106.75 (102.62–112.25)	99.50 (95–106.5)^a^	109.50 (104.62–115.37)	101 (97–110.5)^a^	103.25 (98.25–111.25)	97.50 (88.37–110)^a^	103.75 (100.37–107.75)	98.50 (93–103)^a^	0.010	0.081
HC	112.50 (107.87–122.75)	110.50 (105.5–119)^a^	117.25 (111.37–125.87)	113 (107.−120)^a^	115(106.5–118.62)	109.75 (105.87–115)^a^	111.75 (108.25–116.37)	109 (106.−115.50)^a^	0.221	0.671

Note

Normal Distributed Value are expressed as mean ± *SD*.

Skewed distributed value are expressed with Median (quartile1‐quartile3).

Group 1: plain yogurt, Group 2: probiotic yogurt, Group 3: vitamin D‐fortified yogurt, Group 4: probiotic and vitamin D co‐fortified yogurt.

Within group changes were assessed by Wilcoxon and independent *t*‐test.

Abbreviation: BF, Body Fat; PFB, Percent Body Fat; TBW, Total Body Water; BMI, Body Mass Index; LBM, lean Body Mass; WC, Waist Circumference; HC, Hip Circumference.

^a^ *p*‐value less than .05.

^b^ *p*‐value less than .001.

* Stands for significance of between‐group difference before the intervention (obtained from Kruskal–Wallis test).

** stands for significance of between‐group difference after the intervention (obtained from Kruskal–Wallis test).

### 3.3 Energy and macronutrient intake

The result of calorie intake, percent of each macronutrient intake and dietary intake of vitamin D is shown is Table 4. Carbohydrate intake decline over 10‐wk in group 1 was 5% (*p* = .001), whereas this reduction was 0.6% in group 2 (*p* = .003), 2.1% in group 3 (*p* = .001) and 0.2% in group 4 (*p* = .001) at the end of 10 weeks. However, no difference was seen between groups.

**Table 4:** Mean and Standard deviation of energy and macro nutrient and dietary intake of vitamin D and amount of sun exposure in groups, before and after the intervention

Variable/Yogurt	Group 1 (*n* = 31)		Group 2 (*n* = 28)		Group 3 (*n* = 30)		Group 4 (*n* = 30)		P‐V*	P‐V**
	Before	After	Before	After	Before	After	Before	After	Before	After
MET (min/day)	0 (0–360)	0 (0–1021.38)	384 (0–1668)	1,440 (523–2394)	408 (0–970)	608 (0–1680)	399 (0–960)	372 (0–2274)	0.168	0.058
Sun exposure (min/day)	30 (10–60)	25 (10–30)	30 (10–60)	15 (7.50–30)	60 (13.75–97.50)	25 (15–60)	30 (10–105)	22.50 (10–42.5)	0.649	0.549
Energy (kcal)	1863 (1745–2423)	1551 (1395–1757)^b^	2,220 (1912–2754)	1,820 (1266–2189)^a^	2048 (1648–2759)	1,630 (1293–1928)^b^	2,799 (2109–3096)	1867 (1392–2732)^b^	0.595	0.430
Protein (g/day)	75.89 ± 32.06	61.06 ± 17.04 ^a^	109.86 ± 80.68	71.86 ± 27.98 ^a^	92.17 ± 55.3	75.60 ± 50.69 ^b^	102 ± 33.55	77.84 ± 33.89 ^b^	0.595	0.012
Carbohydrate (g/day)	297.21 ± 93.81	259.49 ± 122.59^a^	340.09 ± 181.09	262.61 ± 189.27^b^	340.09 ± 181.09	262.61 ± 189.27^b^	372.22 ± 142.93	286.35 ± 143.54^b^	0.105	0.153
Fat (g/day)	67.32 ± 16.30	60.04 ± 13.81	49 ± 40.10	40.84 ± 33.78^a^	80.51 ± 34	66.91 ± 28.68^a^	92 ± 22.5	71.70 ± 27.85^a^	0.001	0.019

Note

Normal Distributed Value are expressed as mean ± *SD*.

Skewed distributed value are expressed with Median (quartile1‐quartile3).

Group 1: plain yogurt, Group 2: probiotic yogurt, Group 3: vitamin D‐fortified yogurt, Group 4: probiotic and vitamin D‐fortified yogurt.

Within group changes were assessed by Wilcoxon and independent *t*‐test.

Abbreviation: MET, metabolic equivalent task.

^a^ *p*‐value less than .05.

^b^ *p*‐value less than .001.

* Stands for significance of between‐group difference before the intervention (obtained from Kruskal–Wallis test).

** Stands for significance of between‐group difference after the intervention (obtained from Kruskal–Wallis test).

Fat intake reduction was 2% in group 2 (*p* = .030) and 1.1% in group 3 (*p* = .024) and 1% in group 4 (*p* = .001), whereas it did not change in group 1 (*p* = .084) at the end of 10 weeks. Protein intake decreased by 0.8% (*p* = .011) in group1 compared with 0.4% in group 3 (*p* = .005), whereas it increased by 2.1% in group 2 (*p* = .010) and 0.2% in group 4 (*p* = .001) at the end of 10 weeks.

Regarding dietary intake of vitamin D, the change was not significant neither between nor within groups. Furthermore, comparison changes between groups (Table 4) did not show any significant difference. In addition, after adjusting the confounding effects of BMI, WC, fat and protein, the results remained nonsignificant.

### 3.4 Lipid profile and vitamin D

Result of lipid profile is shown in Table 5. Regarding TG, a significant decrease was seen in all of intervention groups (groups 2, 3, 4), whereas on the other hand, there was no significant difference between groups. Regarding lipid profile LDL, HDL and TC, only probiotic group showed a significant decrease, whereas the difference between groups was not significant. Furthermore, after adjusting confounding effects of BMI, WC, fat and protein, the results remained nonsignificant. The data shown in Table 2 is the result of a total of just 78 participants (26 subjects of group 1, 19 subjects of group 2, 17 subjects of group 3, 15 subjects of group 4.

**Table 5:** lipid profile and vitamin D level of participant before and after the intervention

Variable/Yogurt	Group 1 (*n* = 31)		Group 2 (*n* = 28)		Group 3 (*n* = 30)		Group 4 (*n* = 30)		P‐V*	P‐V**
	Before	After	Before	After	Before	After	Before	After	Before	After
TG (mmol/l)	193.41 ± 70.82	174.75 ± 61.62	226.53 ± 94.07	153.47 ± 46.81	173.20 ± 76.26	143.31 ± 49.81	223.71 ± 65.37	181.62 ± 54.81	0.126	0.210
HDL(mmol/l)	233 ± 45.03	227.08 ± 44.62	254.47 ± 59.20	211.87 45.21	240.40 ± 57.17	22.54 ± 45.18	232.07 ± 61.69	212.62 ± 48.96	0.656	0.438
LDL(mmol/l)	133.76 ± 33.63	128.04 ± 31.45	141.93 ± 47.07	123.20 ± 42.44	137.93 ± 48.05	129.46 ± 40.91	126.62 ± 51.47	108.08 ± 34.60	0.827	0.400
TC (mmol/l)	63.18 ± 11.05	63.71 ± 11.94	63.27 ± 10.87	57.53 ± 6.72	67.53 ± 12.99	65.08 ± 11.62	62.86 ± 14.94	61.92 ± 13.65	0.687	0.289
Vitamin D (µg/l)	32.96 ± 11.92	37.26 ± 14.12	34.78 ± 15.64	36.67 ± 15.79	28.46 ± 14.99	38.56 ± 15.44	30.44 ± 13.01	36.01 ± 11.05	0.415	0.865

Note

*p*‐value < .05 was considered as significant level of differences.

Group 1: plain yogurt, Group 2: probiotic yogurt, Group 3: vitamin D‐fortified yogurt, Group 4: probiotic and vitamin D‐fortified yogurt.

Within group changes were assessed by Wilcoxon and independent *t*‐test.

Abbreviation: TG, Triglyceride; HDL, High Density Lipoprotein; LDL, Low Density Lipoprotein; TC, Total cholesterol.

* Stands for significance of between‐group difference before the intervention (obtained from Kruskal–Wallis test).

** Stands for significance of between‐group difference after the intervention (obtained from Kruskal–Wallis test).

Vitamin D concentration increased significantly in group1 (*p* = .001), group 3 (*p* = .001) and group 4 (*p* = .008), whereas it did not change in group 2 (*p* = .201). However, no difference was seen between groups. Furthermore, comparison changes between groups did not show any significant difference. In addition, after controlling the effects of BMI, WC, fat and protein as confounders, the results did not change.

Gender and age control were done for serum level of vitamin D and after layering for gender and age there was not any difference between groups regarding gender and age distribution based on Pearson chi‐square test (Table 2).

### 3.5 Effect size

The effect size (ES) of low‐calorie diet on calorie intake for plain, probiotic, vitamin D and probiotic and vitamin D cofortified yogurt were 21.65%, 23.48%, 18.73% and 21.43%, respectively. The difference between groups was not significant (*p* = .906). Moreover, regarding weight, there were no differences between 4 yogurts effect size (*p* = .891) (Table 6).

**Table 6:** Effect size of intervention on main outcome

Variable/Yogurt	Group 1 (*n* = 31)	Group 2 (*n* = 28)	Group 3 (*n* = 30)	Group 4 (*n* = 30)	P‐V*
Weight ES	1 (‐0.15–3.02)	1.5 (0.07 ± 4.75)	1.20 (−0.45 ± 3.20)	1.20 (0.02 ± 2.90)	0.891
Vitamin D ES	0 (0 ± 0.25)	0 (0 ± 0)	0 (0 ± 1)	0 (0 ± 1)	0.015

Note

*p*‐value < .05 was considered as significant level of differences.

Values are expressed with Median (quartile1‐quartile3).

Group 1: plain yogurt, Group 2: probiotic yogurt, Group 3: vitamin D‐fortified yogurt, Group 4: probiotic and vitamin D‐fortified yogurt.

Abbreviation: ES, effect size.

* Stand for significance of between‐group difference (obtained from Kruskal–Wallis test).

There was a significant difference between group's ES for serum vitamin D (*p* = .015). Pairwise comparison showed that there was a significant difference in group 1 versus group 3 (*p* = .018) and group 2 versus group 3 (*p* = .002). Vitamin D‐fortified yogurt had the most effect size on serum level of vitamin D; after that vitamin D and probiotic cofortified yogurt, plain and probiotic yogurt, respectively.

## 4 DISCUSSION

Our result showed that serum level of 1, 25(OH)_2_ D_3_ in group 3 increased significantly compare to group 1 and 2. Whereas, there was no significant difference between group 3 and 4. Indeed, vitamin D and probiotic cofortified yogurt might not be superior to vitamin D‐fortified yogurt with regard to increasing serum vitamin D concentration. It was indicated for the first time by L. Jones (Jones et al., 2013), that *Lactobacillus ruteri* increased the serum level of vitamin D. The proposed mechanism behind this is that probiotic might enhance VDR expression and activity, consequently uptake of this vitamin can be increased by enterocytes. Since probiotics behave differently, the difference between our result and L. Jones study might be due to the difference of probiotic strains.

To sum up, our finding show that consumptions of yogurt fortified with 1,000 IU vitamin D in obese subjects with insufficient concentrations at baseline had no significant effect on weight or fat loss and vitamin D status compare to the other groups. Our result is similar to C. Mason, L et al (Mason et al., 2014) who reported no difference in vitamin D status, weight and fat loss in obese people who received 3,320 IU vitamin D_3_ for 12 months while participating in a weight loss program (Jorde et al., 2010).

Based on previous study, this result might be attributed to the fact that, to achieve repletion in obese people, there might be need to supplement higher dose of vitamin D rather than dose prescribed in normal weight (4). In contrast, Salehpour et al. (2012) reported improved vitamin D status and fat mass reduction in 77 obese participants over 12 weeks vitamin D supplementation with 25 µg/day.

TG significantly decreased in all groups with no difference between groups, therefore this result could be pertinent to low‐calorie diet effect. Significant decrease in LDL, HDL and TC was limited to probiotic group, although with no difference between groups. In contrast to our result, Ivey et al. (Madjd et al., 2016) showed that consumption of probiotic yogurt fortified with the same strains for 6 weeks did not change HDL, LDL and TC. On the other hand, our finding is partly consistent with result of a meta‐analysis which reported significant improvement in LDL and TC (Wang et al., 2018), whereas inconsistent about HDL and TG (Ivey et al., 2015). Furthermore, considering the effect of vitamin D supplementation on lipid profile, our finding is consistent with Shah et al. (2015) who reported that large doses of ergocalciferol supplementation at 150,000 IU every 3 months failed to increase not only vitamin D but also lipid profile.

Lipid profile response to exercises is varied based on type, intensity and frequency. Some studies have reported decrease in total cholesterol and an increase in HDL‐ C in moderate and intense types of exercises (Kannan et al., 2014; Kim et al., 2019). However, not all studies have found changes in lipid profile with exercises (Rad & Gholami, 2010). Considering that there was not any difference between four groups regarding physical activity, it seems that in our study weight loss mostly was due to combined effects of exercise and diet and exercise alone was insufficient to stimulate change in any lipid or lipoprotein measures. We have to admit that one of the limitations of our study is that evolution of sun exposure was based on self‐report, on the other hand, the study was done in winter and in a city where the sun exposure is in the least amount in this season.

## 5 CONCLUSIONS

In conclusion, our results showed no positive effect of probiotic consumption on vitamin D absorption. Since probiotic effects are strain specific, our result might be different due to the fact that strains were used in our study are different from previous study. Further studies are needed with other strains of probiotics to reach a consistent result.

## CONFLICT OF INTEREST

The authors declare that they do not have any conflict of interest.

## ETHICAL APPROVAL

The trial received ethical approval from Human Research Ethics Committee of the University of the Medical University of Kermanshah (KUMS.REC1395467). The trial was registered at Iranian registry of clinical trials (IRCT) with number IRCT20120525009856N7.

## Data Availability

The data that support the findings of this study are available from the corresponding author.
